# Biomarkers in predicting mortality among ICU patients with sepsis

**DOI:** 10.1097/MD.0000000000048284

**Published:** 2026-04-10

**Authors:** Sümeyye Uğur, Mustafa Kaçmaz

**Affiliations:** a Department of Anesthesiology and Intensive Care Medicine, Ömer Halisdemir University, Niğde Training and Research Hospital, Niğde, Turkey.

**Keywords:** biomarkers, intensive care unit, mortality, sepsis

## Abstract

Sepsis is one of the most challenging conditions in intensive care units (ICUs), with an increased incidence and mortality, making it critical to predict both mortality and timing of resuscitation. This retrospective study aimed to analyze the association of a number of routinely used biomarkers with mortality in patients admitted to the ICU with sepsis. Patients >18 years of age, who had been admitted to the ICU with sepsis were included. At admission, baseline levels of C-reactive protein (CRP), procalcitonin (PCT), albumin and lactate, as well as white blood cell (WBC), neutrophil, and lymphocyte counts were recorded. The primary outcome was 28-day mortality. Of 235 patients (92 males; mean age 73.57 ± 13.63 years), 28-day mortality occurred in 113 patients (48.1%). The non-survivors differed significantly from survivors with higher WBC, neutrophil and lymphocyte counts, higher levels of PCT, CRP and lactate, and a lower albumin level (for all, *P* < .05). Mortality was associated with lactate, and inversely associated with albumin levels. In logistic regression analysis, albumin and lactate were risk factors to predict 28-day mortality, with odds ratios of 1.51 and 0.89, respectively. In receiver operating characteristic analysis, PCT, lactate and albumin had the greatest values for area under the curve (0.709, 0.654, and 0.696, respectively). Besides established but time-consuming and complicated scoring systems, lactate and albumin levels may be used to predict prognosis in ICU settings, with the advantage of ease in their availability.

## Graphical abstract

**Figure:**
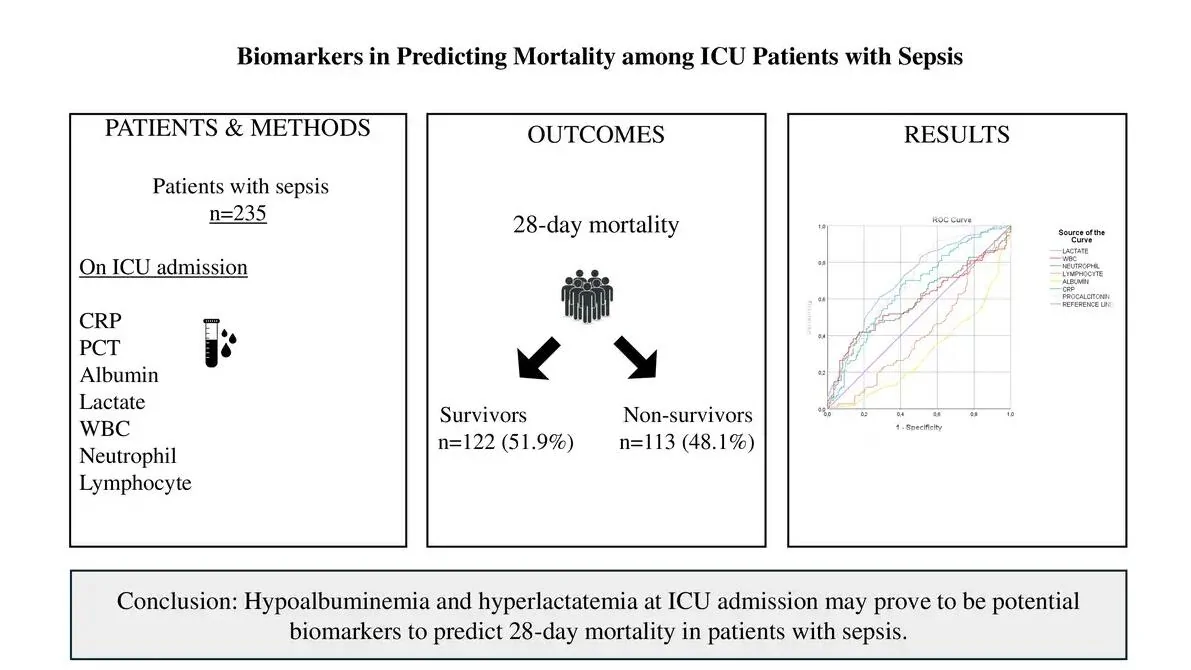

## 1. Introduction

Infectious diseases and sepsis are the leading causes of mortality in intensive care units (ICUs), requiring treatment that is likely to be challenging and long-lasting. Despite advances in early diagnosis and treatment, sepsis remains one of the most important conditions seen in ICUs owing to aging populations and their increasing number of comorbidities. A study reported 48.9 million cases of sepsis and 11 million related deaths annually, with varying incidences and mortality rates worldwide.^[1]^ Given these striking figures, it is of crucial importance to evaluate the prognoses of patients with sepsis on the day of ICU admission, which may be facilitated by readily available biomarkers. Just as their value in diagnosing infectious diseases and monitoring treatment, biomarkers may well be used to predict prognosis in this setting. Risk scoring systems used in predicting disease severity and mortality of ICU patients involve little or no utilization of biomarkers of infection. The Sequential Organ Failure Assessment (SOFA) score includes no biomarkers, while the Acute Physiologic Assessment and Chronic Health Evaluation (APACHE) II scoring system utilizes only the white blood cell (WBC) count. To our knowledge, there have been few studies assessing the role of biomarkers as predictors of prognosis and mortality in ICU patients with sepsis.

This study aimed to evaluate the value of biomarkers, including C-reactive protein (CRP), procalcitonin (PCT), albumin and lactate levels as well as WBC, neutrophil and lymphocyte counts in predicting prognoses of patients with sepsis on the day of ICU admission with their association with 28-day mortality, and to provide more data on the predictive value of biomarkers for mortality as addressed in few previous studies.

## 2. Materials and methods

### 2.1. Patients

The study was approved by the noninvasive Clinical Research Ethics Committee of Niğde Ömer Halisdemir University (approval number 2023/32). This retrospective study included patients >18 years of age, who had been admitted to the ICU with sepsis from May 2022 to May 2023.

Data were obtained from hospital electronic medical records. Patients’ files were reviewed based on the International Classification of Disease-10 (ICD-10). Those whose diagnoses of sepsis were consistent with their epicrises were included. Sepsis was diagnosed based on the Third International Consensus Definitions for Sepsis and Septic Shock.^[2]^ Data included age, sex, duration of ICU stay, site of infections (urinary, abdominal, pulmonary, soft tissue, central nervous system, catheter-related, mixed or others), and comorbidities (hypertension, diabetes mellitus, chronic obstructive pulmonary disease, chronic renal failure, heart failure, coronary artery disease, malignancies, etc). Whether a patient was admitted to the ICU following a scheduled surgery or emergency surgery was also recorded.

Exclusion criteria included any of the following: ICU admission following cardiopulmonary resuscitation or cardiac surgery, a positive test for severe acute respiratory syndrome coronavirus 2 infection, or interruption of treatment because of transfer to another healthcare facility. Patients with missing data were also excluded.

At admission, baseline levels of PCT, CRP, albumin and lactate as well as WBC, neutrophil and lymphocyte counts were obtained. Normal reference range was 0.1 to 2 ng/mL for PCT, 0 to 5 mg/L for CRP, 4 to 10 × 10^3^/µL for WBCs, 2 to 7 × 10^3^/mcL for neutrophils, 0.8 to 4 × 10^3^/mcL for lymphocytes, 3.5 to 5.2 g/dL for albumin, and 0.5 to 1.6 mmol/L for lactate.

The primary outcome was 28-day mortality. Patients were assessed in 2 groups as survivors and non-survivors. For patients with multiple episodes requiring ICU admission, only the most recent episode was taken into consideration.

### 2.2. Statistical analysis

Statistical analysis was performed using the SPSS version 21.0 software (IBM Corp., Armonk). Continuous data were expressed as mean ± standard deviation (SD), while categorical variables were expressed as median and interquartile range or number and frequency. The Student *t* test was used for normally distributed numerical variables, the Mann–Whitney *U* test for non-normally distributed numerical variables and the chi-squared test for categorical variables. Based on the primary outcome, univariate analysis was performed taking 28-day mortality as the dependent variable. Binary logistic regression analysis was used to identify mortality-related markers. Receiver operating characteristic (ROC) analysis was made to identify the value of markers to predict mortality.

## 3. Results

During the study period, a total of 1658 patients were admitted to the ICU, of whom 257 (15.5%) were identified to have primary diagnoses of sepsis at ICU admission. Of these, 22 were excluded from the analysis because of ICU admission after cardiopulmonary resuscitation (n = 13) or missing data (n = 9). Of 235 eligible patients, 143 (61%) were male and 92 (39%) were female. The mean age was 73.6 ± 13.6 years. The mean length of ICU stay was 8.5 ± 10.4 days.

Mortality occurred in 113 patients (48.1%) at 28 days. The mean age was 71.9 ± 13.2 years among survivors and 75.3 ± 13.9 years among non-survivors (*P* > .05). Table 1 shows clinical and laboratory characteristics of survivors and non-survivors.

**Table 1:** Clinical and laboratory characteristics of survivors and non-survivors.

	Total (n = 235)	Survivors (n = 122)	Non-survivors (n = 113)	*P*
Age (yr), mean ± SD	73.57 ± 13.63	71.9 ± 13.2	75.3 ± 14.0	.025
Gender, n (%)				
Female	92 (39%)			
Male	143 (61%)			
ICU days, mean ± SD	8.45 ± 10.42	7.9 ± 11.6	9.1 ± 8.0	**.005**
Comorbid conditions, n (%)				
Hypertension	41 (17.5)	19 (15.6)	22 (19.5)	.432
Diabetes mellitus	31 (13.2)	14 (11.5)	17 (15.0)	.419
COPD	69	47	22	**.001**
Renal failure	28	12	16	.307
Heart failure	36	18	18	.803
CAD	25	12	13	.679
Postoperative ICU	29	8	21	**.005**
ICU after emergency surgery	23	7	16	**.030**
Infection site, n (%)				
Pulmonary	159 (67.7)	85 (69.7)	74 (65.5)	.493
Urinary	27	12	15	.409
Abdominal	38	14	24	**.042**
Soft tissue	13	6	7	.669
Catheter-related	3	2	1	.529
Mixed	10	2	8	.052
Laboratory parameters, mean ± SD				
Procalcitonin (ng/mL)		6.6 ± 19.5	16.0 ± 29.7	**<.001**
C-reactive protein (mg/dL)		115.0 ± 102.5	167.1 ± 105.2	**<.001**
WBC count (10^3^/µL)		11.9 ± 6.2	14.9 ± 10.0	**.039**
Neutrophil count (10^3^/mcL)		10.8 ± 9.7	14.0 ± 2.1	**.016**
Lymphocyte count (10^3^/mcL)		1.7 ± 3.8	1.3 ± 3.4	**.030**
Lactate (mmol/L)		1.9 ± 1.2	3.6 ± 3.5	**<.001**
Albumin (g/L)		32.4 ± 6.8	27.6 ± 6.9	**<.001**

Bold values indicates statistical significance.

CAD = coronary artery disease, COPD = chronic obstructive pulmonary disease, ICU = intensive care unit, SD = standard deviation, WBC = white blood cell.

Based on the site of infection, the majority of patients (n = 159, 67.7%) had respiratory tract infections, followed by abdominal (n = 38), urinary tract (n = 27), soft tissue (n = 13), mixed (n = 10), and catheter-related infections (n = 3). The non-survivor group showed a higher incidence of abdominal infections (*P* = .042; Table 1).

Among the etiologies at ICU admission, chronic obstructive pulmonary disease was most common at 29.4%, with a significantly higher rate in the survivor group (*P* < .001). The rates of ICU admission after surgery and after emergency surgery were significantly higher in the non-survivor group (Table 1).

### 3.1. Laboratory parameters

The non-survivor group had significantly higher WBC, neutrophil and lymphocyte counts, and significantly higher levels of PCT, CRP and lactate, as well as a significantly lower level of albumin (for all, *P* < .05).

In correlation analysis, PCT was moderately correlated with CRP, lactate and albumin (*r* = 0.53, *r* = 0.44, *r* = 0.36, respectively, *P* < .001). A moderate inverse correlation was found between CRP and albumin (*r* = −0.39, *P* < .001; Table 2).

**Table 2:** Results of correlation analysis.

	CRP	WBCs	Neutrophils	Lymphocytes	Lactate	Albumin
PCT	***R*: 0.536** *P*: <.001	*R*: 0.120 *P*: .069	*R*: 0.160 *P*: .015	*R*: −0.254 *P*: <.001	***R*: 0.449** *P*: <.001	**R: 0.369** *P*: <.001
CRP		*R*: 0.039 *P*: .551	*R*: 0.088 *P*: .179	*R*: −0.185 *P*: .005	*R*: 0.218 *P*: .001	***R*: −0.391** *P*: <.001
WBCs			*R*: 0.930 *P*: <.001	*R*: 0.234 *P*: <.001	*R*: 0.071 *P*: .278	*R*: 0.119 *P*: .070
Neutrophils				*R*: 0.121 *P*: .064	*R*: 0.144 *P*:.028	*R*: 0.080 *P*: .222
Lymphocytes					*R*: 0.095 *P*: .147	*R*: 0.121 *P*: .065
Lactate						*R*: −0.166 *P*: .011

Bold values indicates statistical significance.

CRP = C-reactive protein, PCT = procalcitonin, WBC = white blood cell.

In binary logistic regression analysis, albumin and lactate were found as predictors of 28-day mortality, with odds ratios of 1.514 and 0.889, respectively (Table 3). Mortality was associated with the lactate level, and showed an inverse association with the albumin level.

**Table 3:** Binary logistic regression analysis of biomarkers in association with 28-day mortality.

	*B*	Exp(*B*)	*P*	Lower	Upper
PCT	−0.004	0.996	.610	0.982	1.011
CRP	0.002	1.002	.227	0.999	1.005
WBCs	0.071	1.073	.016	1.014	1.137
Neutrophils	−0.006	0.994	.775	0.952	1.038
Lymphocytes	−0.092	0.912	.095	0.819	1.016
Lactate	0.414	**1.514**	**<.001**	1.212	1.891
Albumin	−0.118	**0.889**	**<.001**	0.844	0.937

Bold values indicates statistical significance.

CRP = C-reactive protein, PCT = procalcitonin, WBC = white blood cell.

**Figure 1.**
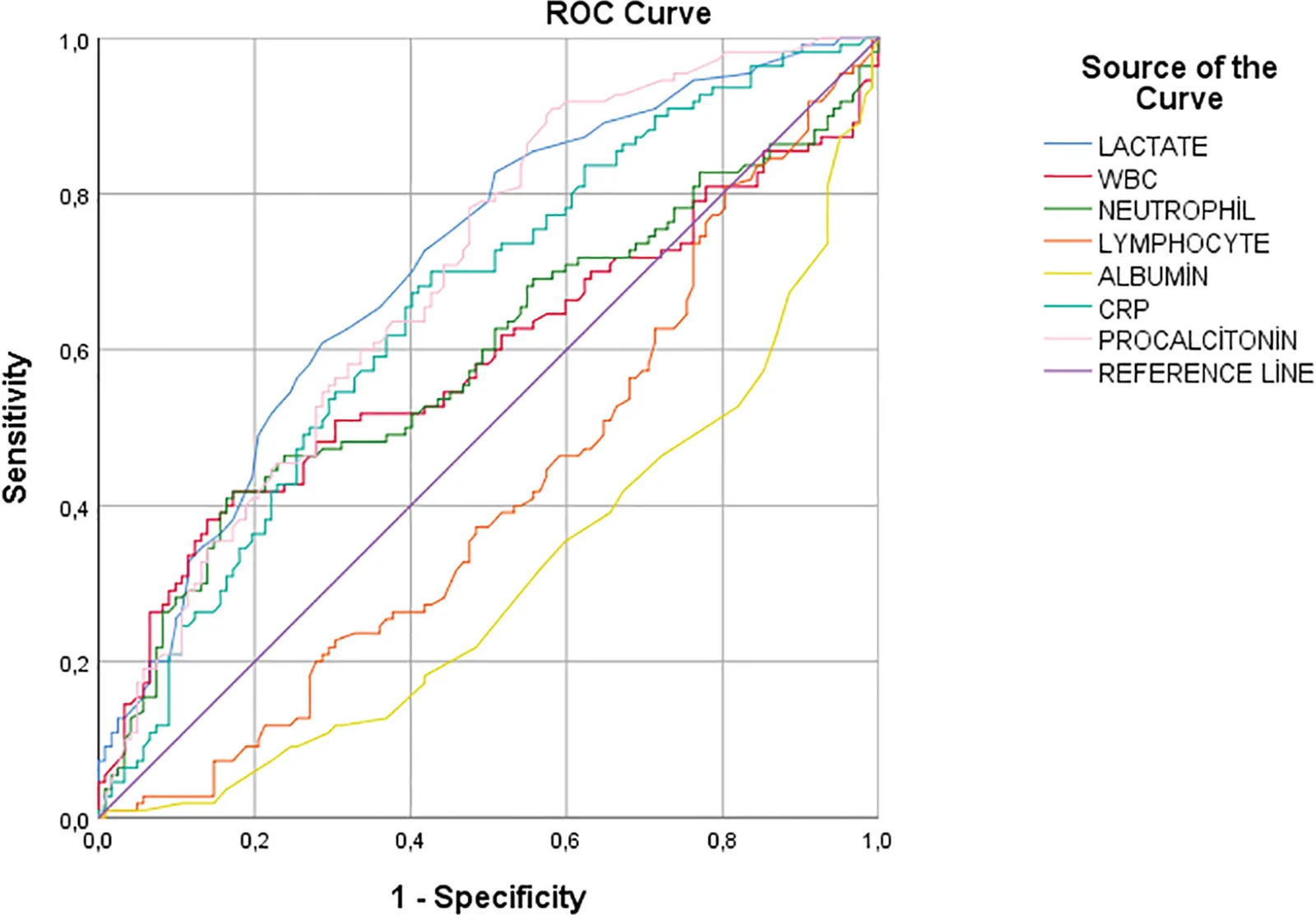
ROC curves of biomarkers concerning 28-day mortality. CRP = C-reactive protein, ROC = receiver operating characteristic, WBC = white blood cell.

In ROC analysis, PCT, lactate and albumin had the greatest areas under the curve (AUC; Table 4 and Fig. 1). The AUC for PCT was 0.709, with a sensitivity of 64.3% and a specificity of 77.5 % (95% CI). The corresponding AUCs for lactate and albumin were 0.654 (sensitivity 58.3%, specificity 72.4 %) and 0.696 (sensitivity 62.9%, specificity 76.2%). The optimal cut-off levels for albumin and lactate were 3.25 g/dL and 1.65 mmol/L, respectively.

**Table 4:** ROC curve analysis of biomarkers in terms of 28-day mortality.

	AUC (95% CI)	Cut-off	*P*	Sensitivity	Specificity
PCT	**0.709**	0.38	<.001	0.643	0.775
CRP	0.584	86.90	.026	0.509	0.660
WBCs	0.590	10.72	.018	0.515	0.665
Neutrophils	0.414	9.18	.024	0.341	0.487
Lymphocytes	0.305	0.95	<.001	0.238	0.372
Lactate	**0.654**	1.65	<.001	0.583	0.724
Albumin	**0.696**	32.50	<.001	0.629	0.762

Bold values indicates statistical significance.

AUC = area under the curve, CRP = C-reactive protein, PCT = procalcitonin, ROC = receiver operating characteristic, WBC = white blood cell.

### 3.2. Post-hoc power analysis

The mean lactate levels were 3.60 ± 3.46 mmol/L in the non-survivor group (n = 113) and 1.94 ± 1.24 mmol/L in the survivor group. The 2-tailed Welch *t* test showed a mean difference of 1.66 mmol/L between the groups (95% CI: 0.98–2.34), *t* (138.4) = 4.82, *P* < .001; Cohen *d* = 0.65.

The mean albumin levels were 27.6 ± 6.9 g/dL in the non-survivor group and 32.4 ± 6.8 g/dL in the survivor group. The 2-tailed Welch *t* test showed a mean difference of−4.80 g/dL between the groups (95% CI: −6.55 and−3.05), *t* (232) = −5.39, *P* < .001; Cohen *d* = 0.71.

## 4. Discussion

The current study investigated the role of biomarkers in predicting 28-day mortality among ICU patients with sepsis and found that lactate and albumin levels at the time of ICU admission enabled prediction of 28-day mortality. Our results showed that the non-survivor group had significantly higher levels of lactate and lower levels of albumin, in logistic regression analysis, lactate and albumin were risk factors for mortality, and in ROC analysis, albumin and lactate levels discriminated moderately well between survivors and non-survivors, with cut-off values of 1.65 mmol/L and 3.25 g/dL, respectively.

In routine practice, the SOFA score is used to evaluate mortality in ICU patients with sepsis. It mainly assesses organ dysfunction; however, alternative methods may be used for earlier identification of infection that would increase the risk for organ dysfunction. Our results suggest that, rather than time-consuming and complicated scoring systems, easily available biomarkers, that is, lactate and albumin, may be conveniently used to predict prognosis.

In critically ill patients, rather than malnutrition, hypoalbuminemia is more likely to be associated with physiologic stress caused by disease or acute trauma. A randomized controlled study found that, while albumin production did not differ in both septic and non-septic groups, the septic group had lower albumin levels, indicating that the cause of hypoalbuminemia in sepsis is not due to the decreased production of albumin, but due to its increased clearance from the circulation.^[3]^ In the setting of inflammation, albumin escapes to the interstitial area owing to increased capillary permeability, which ends up with a greater distribution volume; hence, ensuing hypoalbuminemia as an indicator of inflammation.^[4]^ The severer the inflammation is, the lower the level of albumin,^[5]^ This inverse relationship between albumin and inflammation may enable the evaluation of both prognosis and the efficacy of treatment. Therefore, inflammation-induced hypoalbuminemia may be hypothesized to be a predictor for poor survival. Hypoalbuminemia has been shown to be indicative of mortality among patients with sepsis or suspected sepsis. A retrospective study from China involving 5357 patients with sepsis found, with a cut-off level of 2.6 g/dL, that albumin levels at ICU admission were associated with both short-term (28-day/60-day) and long-term (180-day/1-year) mortality rates. Furthermore, an increase of 1 g/dL in the albumin level resulted in a risk reduction by 39% in 28-day mortality. Conversely, a decrease of 1 g/dL in the albumin level below the cut-off value (2.6 g/dL) increased the risk by 59%.^[6]^ In another study of 577 patients with sepsis, the serum albumin level at ICU admission predicted mortality with a cut-off value of 2.45 g/dL, and a decrease of 1 g/dL below 2.45 g/dL increased the risk by 63.4%.^[7]^ In sepsis patients aged 65 years or older, an albumin level of <2.6 g/dL at admission to the emergency department (ED) was found to be a robust predictor for 30-day mortality.^[8]^ In the present study, the non-survivor group had a significantly lower albumin level and, in binary logistic regression analysis, albumin was found to be a predictor of 28-day mortality. In ROC analysis, the cut-off value for albumin was estimated at 3.25 g/dL.

Some studies also assessed the albumin level in relation to the SOFA score. A prospective study of 962 patients presenting to the ED with infection found hypoalbuminemia to be a predictor for 30-day mortality, with the AUC being greater in patients with a SOFA score of <2.^[9]^ In another study, the same authors even proposed that the ability of hypoalbuminemia to predict mortality was similar to that of the SOFA score. In particular, the precision of prediction was highest among patients with a SOFA score of <2.^[10]^ In such settings, hypoalbuminemia may allow early identification of microvascular alterations that have not yet manifested themselves in organ systems to be evaluated with the SOFA score.

The other biomarker that was found to predict mortality in ICU patients with sepsis was the lactate level, which showed an inverse relationship with the albumin level. Increases in lactate levels are commonly accepted to reflect tissue hypoperfusion caused by anaerobic glycolysis as well as insufficient oxygen delivery to the tissues. However, this may not be the case in sepsis patients with hyperlactatemia, because in these patients, oxygen delivery to the tissues is often adequate owing to a hyperdynamic circulation. The current opinion is that endogenous epinephrine is released in response to sepsis-related stress, causing increased lactate levels.^[11]^ Beta-adrenergic stimulation provokes glycolysis through which overproduced pyruvate turns into lactate, increasing lactate levels, which then becomes an energy source for vital organs in the setting of stress.^[12]^ Accordingly, given that hyperlactatemia is a sign of increased endogenous epinephrine, it may herald a shock that is concealed by endogenous epinephrine in patients with no hypotension. Therefore, the coexistence of sepsis and hyperlactatemia may represent an increased risk for mortality.

Based on a subset analysis of the Surviving Sepsis Campaign database, lactate levels higher than 4 mmol/L in the presence or absence of hypotension were highly associated with in-hospital mortality. In the presence of hypotension, a lactate level of more than 2 mmol/L was shown to increase mortality.^[13]^ A large retrospective study from Spain investigated the relationship between hyperlactatemia (≥5 mmol/L) and in-hospital mortality in ICU patients. Increased lactate levels were found to be associated with mortality, with the optimal cut-off value varying based on the etiology, that is, being the lowest (6.2 mmol/L) following cardiac surgery and the highest following cardiac arrest (11.8 mmol/L).^[14]^ A Canadian study found that higher lactate levels (1.4–2.3 mmol/L), even within the normal range, predicted 28-day mortality as well as organ dysfunction in patients with septic shock.^[15]^ A retrospective study of 3325 patients with or without sepsis found an association between increased lactate levels and short- (3 days) and long-term (30 days, 1 year) mortality. The risk for short-term mortality increased with a lactate level of higher than 4 mmol/L (normal range < 0.4 mmol/L), whereas slightly elevated lactate levels (0.4–4 mmol/L) were suggestive of long-term mortality.^[16]^ In a study of 830 patients presenting to the ED with severe sepsis, baseline both moderately (2–3.9 mmol/L) and highly elevated (≥4 mmol/L) lactate levels were associated with 28-day mortality.^[17]^ In another study, baseline lactate levels of <4 mmol/L were found to be an independent risk factor for in-hospital mortality in patients with septic shock.^[18]^ In the current study, a cut-off value of 1.65 mmol/L for lactate, close to the reference range, discriminated moderately well between survivors and non-survivors.

Like hypoalbuminemia, the lactate level in relation to the SOFA score has also been examined. A prospective study recorded lactate levels and SOFA scores on 0 to 7 days of ICU admission and found strong correlations between the two.^[19]^ A retrospective study of 1865 patients also found lactate as a predictor for both short- and long-term mortality (30-day, 60-day, 1-year), with a similar power to that of the SOFA score.^[20]^ In a prospective observational study from Spain conducted among patients with sepsis and a SOFA score of ≤6, baseline lactate level was the best predictor for both hospital-acquired infections (AUC 0.70) and all-cause 28-day mortality (AUC 0.67).^[21]^

Despite being a validated predictor for mortality in sepsis,^[22,23]^ the use of the SOFA score has several limitations, particularly at times when prompt intervention in response to fluctuations in patients’ conditions is required. In such settings, both albumin and lactate levels may reflect worsening at a much earlier time before it has become sufficiently manifest for assessment by the SOFA score.

Apart from albumin and lactate levels, other biomarkers such as CRP, PCT, and neutrophil-to-lymphocyte ratio (NLR) have been examined for their predictive roles in ICU patients with suspected sepsis or sepsis, but in relatively small sample sizes.^[24,25]^ In these analyses, PCT at ICU admission predicted 28-day mortality in 1 study (n = 146),^[24]^ and PCT and CRP at ICU admission predicted 28-day mortality in another (n = 536).^[25]^ However, a meta-analysis found that none of the biomarkers (PCT, CRP, IL-6, or sCD14) examined at baseline had any predictive value for mortality in ICU patients with sepsis,^[26]^ a finding consistent with our results.

Our study has several limitations. It is a single-center study, has a retrospective design and a relatively small sample size. Data on nutritional status and albumin replacement of patients prior to ICU admission were missing. Hepatic or renal failure was not set as an exclusion criterion due to challenges about their definition, so to what extent hyperlactatemia in the presence of hepatic or renal dysfunction would predict mortality could not be assessed.

In conclusion, our findings suggest that hypoalbuminemia and hyperlactatemia at ICU admission may have potential to predict 28-day mortality in patients with sepsis, both of which may also serve as indicators for initiating resuscitation. Since both albumin and lactate measurements are routinely performed at ICU admission, they can guide treatment and interventions as well as provide a rapid insight into the prognosis of sepsis. Further larger studies are required, with adjustment of confounding factors, such as age, postoperative status and comorbidities.

## Acknowledgments

We are thankful to Mrs. Kafiye May and Mr. Hasan May for their assistance with English language and their support.

## Author contributions

**Conceptualization:** Sümeyye Uğur, Mustafa Kaçmaz.

**Data curation:** Sümeyye Uğur, Mustafa Kaçmaz.

**Formal analysis:** Sümeyye Uğur, Mustafa Kaçmaz.

**Funding acquisition:** Sümeyye Uğur, Mustafa Kaçmaz.

**Investigation:** Sümeyye Uğur, Mustafa Kaçmaz.

**Methodology:** Sümeyye Uğur, Mustafa Kaçmaz.

**Project administration:** Sümeyye Uğur, Mustafa Kaçmaz.

**Resources:** Sümeyye Uğur, Mustafa Kaçmaz.

**Software:** Sümeyye Uğur, Mustafa Kaçmaz.

**Supervision:** Sümeyye Uğur, Mustafa Kaçmaz.

**Validation:** Sümeyye Uğur, Mustafa Kaçmaz.

**Visualization:** Sümeyye Uğur, Mustafa Kaçmaz.

**Writing – original draft:** Sümeyye Uğur, Mustafa Kaçmaz.

**Writing – review & editing:** Sümeyye Uğur, Mustafa Kaçmaz.
